# European Society for Paediatric Endocrinology Consensus Guidelines on Screening, Diagnosis, and Management of Congenital Hypothyroidism

**DOI:** 10.1210/jc.2013-1891

**Published:** 2014-01-21

**Authors:** Juliane Léger, Antonella Olivieri, Malcolm Donaldson, Toni Torresani, Heiko Krude, Guy van Vliet, Michel Polak, Gary Butler

**Affiliations:** Université Paris Diderot (J.L.), Sorbonne Paris Cité, F-75019 Paris, France; Assistance Publique-Hôpitaux de Paris (AP-HP), Hôpital Robert Debré, Service d'Endocrinologie Diabétologie Pédiatrique et Centre de Référence des Maladies Endocriniennes Rares de la Croissance, F-75019, Paris, France; Institut National de la Santé et de la Recherche Médicale (INSERM), Unité Mixte de Recherche 676, F-75019 Paris, France; Department of Cell Biology and Neurosciences (A.O.), Istituto Superiore di Sanità, 00161 Rome, Italy; Child Health Section of Glasgow University School of Medicine (M.D.), Royal Hospital for Sick Children, Yorkhill, Glasgow G3 8SJ, Scotland, United Kingdom; Swiss Neonatal Screening Laboratory (T.T.), University Children's Hospital, CH-8032 Zurich, Switzerland; Department of Pediatric Endocrinology and Diabetes (H.K.), Charite Children's Hospital, Berlin 10117, Germany; Endocrinology Service and Research Center (G.v.V.), Centre Hospitalier Universitaire Sainte-Justine and Department of Pediatrics, University of Montreal, Montreal, Canada H3T 1C5; AP-HP, Hôpital Necker Enfants-Malades, Endocrinologie, Gynécologie et Diabétologie Pédiatriques (M.P.), Centre de Référence des Maladies Endocriniennes Rares de la Croissance, Université Paris Descartes, Sorbonne Paris Cité, INSERM, Unité 845, F-75015 Paris, France; and Department of Paediatric and Adolescent Medicine and Endocrinology (G.B.), University College London Hospital, and University College London Institute of Child Health, London NW1 2PQ, United Kingdom

## Abstract

**Objective:**

The aim was to formulate practice guidelines for the diagnosis and management of congenital hypothyroidism (CH).

**Evidence:**

A systematic literature search was conducted to identify key articles relating to the screening, diagnosis, and management of CH. The evidence-based guidelines were developed with the Grading of Recommendations, Assessment, Development and Evaluation (GRADE) system, describing both the strength of recommendations and the quality of evidence. In the absence of sufficient evidence, conclusions were based on expert opinion.

**Consensus Process:**

Thirty-two participants drawn from the European Society for Paediatric Endocrinology and five other major scientific societies in the field of pediatric endocrinology were allocated to working groups with assigned topics and specific questions. Each group searched the literature, evaluated the evidence, and developed a draft document. These papers were debated and finalized by each group before presentation to the full assembly for further discussion and agreement.

**Recommendations:**

The recommendations include: worldwide neonatal screening, approaches to assess the cause (including genotyping) and the severity of the disorder, the immediate initiation of appropriate L-T_4_ supplementation and frequent monitoring to ensure dose adjustments to keep thyroid hormone levels in the target ranges, a trial of treatment in patients suspected of transient CH, regular assessments of developmental and neurosensory functions, consulting health professionals as appropriate, and education about CH. The harmonization of diagnosis, management, and routine health surveillance would not only optimize patient outcomes, but should also facilitate epidemiological studies of the disorder. Individuals with CH require monitoring throughout their lives, particularly during early childhood and pregnancy.

## Summary of Recommendations

### 1.1 The benefits of congenital hypothyroidism screening

- Early detection and treatment of congenital hypothyroidism (CH) through neonatal screening prevents neurodevelopmental disability and optimizes developmental outcomes (1|⊕⊕⊕).

### 1.2 Analytical methodology, effectiveness, and efficacy of CH screening strategies

- Screening for primary CH should be introduced worldwide. The initial priority of neonatal screening for CH should be the detection of all forms of primary CH: mild, moderate, and severe. The most sensitive test for detecting primary CH is TSH determination (1|⊕⊕⊕).

### 1.3 Screening in special categories of neonates at risk of CH

- A strategy of second screening should be considered for the following conditions: preterm neonates; low-birth weight (LBW) and very low-birth weight (VLBW) neonates; ill and preterm newborns admitted to neonatal intensive care units (NICU); specimen collection within the first 24 hours of life; and multiple births (particularly same-sex twins); (2|⊕⊕○).

### 2.1 Biochemical criteria used in the decision to initiate treatment

- If capillary TSH concentration from blood obtained on neonatal screening is ≥ 40 mU/L whole blood, we recommend starting treatment as soon as a good venous sample can be obtained, without waiting for the venous blood test result, unless venous thyroid function test (TFT) results are available on the same day (1|⊕⊕○).
- If capillary TSH concentration is < 40 mU/l of whole blood, the clinician may wait for the results of venous TFT, provided that these results are available on the following day (1|⊕⊕○).

### 2.2 Communication of elevated TSH result

- The detection of a high TSH concentration on screening should be communicated by an experienced person (eg screening laboratory staff or pediatric endocrine team) either by telephone or in person (2|⊕○○).
- When the child reaches nursery or school age, educators and teachers need not be informed about the child having CH to avoid stigmatization due to “labeling” (2|⊕○○).

### 2.3 Decision to start treatment on the basis of venous TFTs

- If venous free T_4_ (FT4) concentration is below norms for age, treatment should be started immediately (1|⊕⊕⊕).
- If venous TSH concentration is > 20 mU/L, treatment should be started, even if FT4 concentration is normal (2|⊕⊕○).
- If venous TSH concentration is ≥ 6 to 20 mU/l beyond 21 days in a well baby with a FT4 concentration within the limits for age, we suggest a) investigation, which should include diagnostic imaging, to try to obtain a definitive diagnosis; b) consideration, in discussion with the family, of either initiating thyroxine supplementation immediately and retesting, off treatment, at a later stage; or withholding treatment but retesting two weeks later (2|⊕⊕○).

### 2.4 Use of imaging in assessing the severity and cause of CH

- X-ray of the knee may be carried out to assess the severity of intrauterine hypothyroidism by the presence or absence of femoral and tibial epiphyses (2|⊕⊕⊕).
- The thyroid gland should be imaged using either radioisotope scanning (scintigraphy) with or without the perchlorate discharge test; or ultrasonography; or both (1|⊕⊕○).
- Imaging should never be allowed to delay the initiation of treatment (1|⊕⊕○).

### 2.5 Associated malformations and syndromes

- All neonates with high TSH concentrations should be examined carefully for congenital malformations (particularly cardiac) and for dysmorphic features (1|⊕⊕⊕).

### 3.1 Treatment and monitoring of CH

- L-T_4_ alone is recommended as the medication of choice for treating CH (1|⊕⊕○).
- L-T_4_ treatment should be initiated as soon as possible and no later than 2 weeks after birth or immediately after confirmatory serum test results in infants in whom CH is detected by a second routine screening test (1|⊕⊕○).
- An initial L-T4 dose of 10–15 μg/kg per day should be given (1|⊕⊕○).
- Infants with severe disease, as defined by a very low pretreatment TT_4_ or FT4 concentration, should be treated with the highest initial dose (1|⊕⊕○).
- L-T_4_ should be administered orally; if intravenous treatment is necessary the dose should be no more than 80% of the oral dose, The dose should then be adjusted according to TSH and FT4 determinations (1|⊕⊕○).
- L-T_4_ tablets should be crushed and administered via a small spoon, in a few milliliters of water or breast milk (1|⊕⊕○).
- Brand rather than generic L-T_4_ tablets should be used, particularly during infancy and in severe cases (2|⊕⊕○).
- L-T_4_ liquid should only be used if pharmaceutically produced (1|⊕⊕○).
- Parents should be provided with written instructions on L-T_4_ treatment (1|⊕○○).

### 3.2 Monitoring of dose and follow-up

- Serum or plasma FT4 (or TT4) and TSH concentrations should be determined at least 4 hours after the last L-T_4_ administration (1|⊕⊕○).
- TSH concentration should be maintained in the age-specific reference range; TT_4_ or FT4 concentration should be maintained in the upper half of the age-specific reference range (1|⊕⊕○).
- Any reduction of L-T_4_ dose should not be based on a single increase in FT4 concentration during treatment (1|⊕⊕○).
- The first follow-up examination should take place 1–2 weeks after the start of L-T_4_ treatment (1|⊕○○).
- Subsequent evaluation should take place every 2 weeks until a complete normalization of TSH concentration is reached; then every 1 to 3 months thereafter until the age of 12 months (1|⊕○○). Between the ages of one and three years, children should undergo frequent clinical and laboratory evaluations (every 2 to 4 months) (1|⊕○○). Thereafter, evaluations should be carried out every 3 to 12 months until growth is completed (1|⊕○○).
- More frequent evaluations should be carried out if compliance is questioned or abnormal values are obtained (1|⊕○○).
- Additional evaluations should be carried out 4–6 weeks after any change in L-T_4_ dose or formulation (1| ⊕○○).
- Adequate treatment throughout childhood is essential and overtreatment should be avoided (1|⊕⊕⊕).

### 3.3 Thyroid re-evaluation

- Re-evaluation of the thyroid axis is indicated when no diagnostic assessment was carried out in infancy, and particularly when the infant was preterm/sick at the time of referral (1|⊕⊕⊕).
- For a precise diagnosis, L-T_4_ treatment should be phased out over a 4- to 6-week period, and a full reevaluation should be carried out, with both biochemical testing and thyroid imaging if hypothyroidism is confirmed (2|⊕⊕○).
- If the presence or absence of primary CH is being assessed, rather than an exact diagnosis being sought, re-evaluation may be carried out by decreasing the dose of L-T_4_ by 30% for 2–3 weeks and then rechecking thyroid function. If an increase in TSH concentrations to ≥ 10 mU/L is demonstrated, CH is confirmed. Otherwise the dose can be reduced further, with retesting after another 2–3 weeks. (2|⊕⊕○).

### 3.4 Treatment and monitoring in pregnant women with CH

- We recommend an immediate increase in L-T4 dose by 25–30% following a missed menstrual cycle or positive home pregnancy test (1|⊕○○).
- TSH and FT4 (or total T_4_ [TT4]) levels should be monitored every 4–6 weeks during the pregnancy, aiming at TSH concentrations < 2.5 mU/L in the first trimester and < 3 mU/L later in pregnancy (1|⊕○○).

### 4.1 Outcomes in treated patients

- Psychomotor development and school progression should be monitored and recorded in all children with CH, and particularly in at-risk cases (absent knee epiphyses at term, very low TT_4_ or FT4, and very high TSH concentrations at diagnosis, athyreosis, delayed normalization of TSH, poor control during the first year, delayed milestones) (1|⊕⊕⊕).
- A personalized educational plan is required if school progress is affected in cases of severe CH (2|⊕⊕○).
- Concerns about behavior should be addressed from the time of diagnosis until school age (2|⊕⊕○).
- Memory deficits may be corrected by targeted training (2|⊕⊕○).
- Repeated (not just neonatal) hearing tests should be carried out before school age and, as required (2|⊕⊕○).
- We recommend assessment for evidence of visual processing problems (not just visual acuity) (2|⊕○○).
- We recommend screening for speech delay and referral for speech therapy by 3 years if required (2|⊕⊕○).

### 4.2 Health-related quality of life (HrQOL)

- Compliance with treatment should be promoted throughout life (1|⊕⊕⊕).
- There is a risk of subtle decrease in HrQOL in young adulthood, particularly if treatment is suboptimal (2|⊕○○).

### 4.3 Patient education and compliance/adherence

- Medical education about CH should be improved at all levels, with regular updates (1|⊕⊕⊕).
- The education of both parents and patients is essential particularly during transition to adult care and during pregnancy (1|⊕⊕⊕).

### 4.4 Growth, puberty, and fertility

- Adherence to treatment influences growth and should be promoted (1|⊕⊕⊕).
- Normal growth, puberty, and fertility can be anticipated, if adherence is reasonably good (1|⊕⊕⊕).

### 4.5 Bone health

- Patients with CH should be adequately treated with thyroxine, consume 800–1200 mg of calcium daily, and receive supplements added if intake is insufficient (2|⊕○○).

### 4.6 Metabolic and cardiovascular health

- We recommend lifestyle interventions, including diet and exercise, to optimize weight and health in individuals with CH (2|⊕○○).

### 5.1 Criteria for genetic counselling

- Genetic counseling should involve explaining the risk of recurrence of CH in an affected family, based on family history and thyroid morphology (1|⊕⊕○).
- Each family with an affected child should have access to information about the two major forms of CH (dysgenesis and dyshormonogenesis) and should if possible receive an explanation of their inheritance and recurrence rate (1|⊕⊕○).
- Genetic counseling should be targeted rather than general (2|⊕⊕○).

### 5.2 Molecular biology in the diagnosis and management of CH

- Molecular genetic study should be preceded by a careful phenotypic description of CH patients (including morphology of the thyroid gland) (1|⊕⊕○).
- Any syndromic association should be studied genetically, to identify new CH genes and to make it possible to provide appropriate genetic counseling (1|⊕⊕○).
- The presence of familial cases of dysgenesis in siblings or parents should lead to a search for TSH receptor or PAX8 mutations, respectively (2|⊕⊕○).

### 5.3 Antenatal diagnosis, screening, and potential treatment of fetal CH

- We recommend antenatal diagnosis when goiter is fortuitously discovered during fetal ultrasound, with a family history of dyshormonogenesis and with known defects of genes involved in thyroid function or development (1|⊕⊕○).
- The therapeutic management of affected fetuses should comply with the laws of the country concerned (1|⊕⊕○).
- Cordocentesis, rather than amniocentesis, should be the reference method for assessing fetal thyroid function but only performed if prenatal intervention is considered (1|⊕⊕⊕).
- In a euthyroid pregnant woman, a large goiter in the fetus with progressive hydramnios and a risk of premature labor and delivery and/or concerns about tracheal occlusion are criteria in favor of fetal treatment in utero (1|⊕⊕○).
- Interventions such as intra-amniotic L-T4 injection should be performed only by multidisciplinary specialist teams (1|⊕⊕⊕).

### Conclusion

Further research is required to improve our understanding of the pathophysiology and management of this heterogenous disorder.

Thyroid hormones play a crucial role in early neurodevelopment so that untreated severe CH results in neurological and psychiatric deficits, including intellectual disability, spasticity, and disturbances of gait and co-ordination. CH is one of the most common preventable causes of mental retardation. Screening programs, which have been in operation over the last 30 years in most industrialized countries, have led to the successful early detection and treatment of infants with CH and have eliminated the severe neurodevelopmental deficits resulting from late diagnosis. Studies on cognitive function in patients with CH treated soon after birth have shown that normal development can be achieved in most patients, although some may have subtle neurocognitive deficits (1).

Estimates of the prevalence of CH vary according to the method of ascertainment: about 1 in 2000 to 3000 live births in countries with neonatal screening vs about 1 in 6700 live births before the screening era (1). Recent reports have indicated that the incidence of primary CH may be increasing in some countries, particularly for cases with a normally located (eutopic) thyroid gland and milder dysfunction. The reasons for this remain unclear (2) but may relate to changes in screening thresholds (3, 4).

The results from neonatal screening programs have also helped to identify a broad spectrum of thyroid dysfunctions with different underlying etiologies. CH can be classified according to site: primary (thyroid) or secondary/central (pituitary and/or hypothalamic); to severity: FT4 levels within the normal range for age (compensated) or subnormal (decompensated); and to age of onset. The most common form of CH is primary hypothyroidism, with high TSH levels reflecting various types of abnormal thyroid gland development or dyshormonogenesis. Secondary hypothyroidism is much less frequent, either with isolated TSH deficiency due to mutations inactivating the TSH β-subunit, the TRH receptor, or IGSF1 (ImmunoGlobulin SuperFamily member 1), or more commonly with TSH deficiency associated with other pituitary hormone deficiencies.

Impaired thyroid hormone production may also be temporary or permanent, the latter requiring lifelong treatment, and thyroid dysfunction may change in a given individual with growth and development stages (5). Transient primary CH can be defined as an increase in TSH levels during the neonatal period, with normal TFT results obtained off treatment at a later stage. The purely descriptive term “hyperthyrotropinemia” refers to a form of compensated CH in which there is a mild increase in TSH concentration (eg, 6–20 mU/L) with normal thyroid hormone concentrations. It may also be transient or permanent.

## Method for Developing Evidence-Based Recommendations

Given the importance of optimal screening, prompt diagnosis, and adequate treatment of CH, and in recognition of the considerable variations in its management worldwide, and the stated need for a consensus-building conference (6), the European Society for Paediatric Endocrinology (ESPE) decided to examine current best practice in CH and to formulate evidence-based recommendations. This was done by convening a panel of experts from the ESPE for a consensus conference on CH and also by inviting participation from members of the following societies: the Pediatric Endocrine Society [North America] (PES); the Asia Pacific Paediatric Endocrine Society (APPES); the Japanese Society for Pediatric Endocrinology (JSPE); the Sociedad Latino-Americana de Endocrinología Pediátrica (SLEP); the Australasian Paediatric Endocrine Group (APEG); and the Indian Society for Pediatric and Adolescent Endocrinology (ISPAE).

The target audience for these guidelines includes general and specialist pediatricians, other professionals providing care for patients with CH, and policy makers, particularly in countries with developing economies currently in the process of initiating neonatal screening programs for CH.

Participants included individuals from Europe, North America (United States and Canada), Latin America, Asia, and Australia, with a balanced spectrum of professional seniority and expertise. In addition, an expert on the development of evidence-based guidelines was recruited to serve in an advisory capacity. Panel members declared whether they had any potential conflict of interest at the initial meeting of the group.

Thirty-two participants were assigned to one of five groups to which topics 1–5 were allocated, and a chairperson was designated for each group. Each participant prepared a summary of the literature relating to a particular question distributed before the conference (which was held over 2 days in November 2011). Each group revised the summaries, which were then presented to the full conference. This report is based on the questions addressed.

A detailed description of the grading scheme has been published elsewhere (7). Recommendations were based on published findings and on expert opinion when appropriate. The best available research evidence was used to develop recommendations. Preference was given to articles written in English, identified by PubMed searches with MeSH terms.

For each point, recommendations and evidence are described, with a modification in the grading evidence as follows: 1 = strong recommendation (applies to most patients in most circumstances, benefits clearly outweigh the risk); and 2 = weak recommendation (consensus opinion of working group or should be considered; the best action may depend on circumstances or patient values, benefits, and risks closely balanced or uncertain).

Qualify of evidence is indicated as follows: ⊕⊕⊕, high quality (prospective cohort studies or randomized controlled trials at low risk of bias); ⊕⊕○, moderate quality (observational studies or trials with methodological flaws, inconsistent or indirect evidence); and ⊕○○, low quality (case series or nonsystematic clinical observations).

## 1.0 Neonatal Screening

### 1.1 The benefits of CH screening

#### Recommendations

1.1.1 Neonatal screening programs for CH have been highly successful and economically beneficial over the last four decades. Affected children are detected very soon after birth, mostly before clinical symptoms and signs become evident. Early detection and treatment prevent morbidity, particularly neurodevelopmental disabilities (1|⊕⊕⊕).

#### 1.1.1 Evidence

Many studies have confirmed the early success of CH screening for normalizing the cognitive outcomes of children with severe primary CH (8, 9), and the timing of the normalization of thyroid function may influence the outcome (10). The avoided lifetime costs of care for children in whom intellectual disability is prevented as a result of screening for CH have been estimated to exceed the costs of screening and diagnosis by a large margin (11).

### 1.2 Analytical methodology, effectiveness, and efficacy of CH screening strategies

#### Recommendations

1.2.1 Screening for primary CH worldwide should be performed wherever possible on the basis of national resources. For new programs, there is a need to decide on the scope of screening to define the strategy for selecting neonatal screening tests. The goal of neonatal screening should be to detect all forms of primary CH—mild, moderate, and severe—but particularly those patients with severe CH in whom morbidity is high. The most sensitive test for detecting primary CH is the determination of TSH concentration (1|⊕⊕⊕).

1.2.2 Primary CH screening has been shown to be effective for the testing of cord blood or blood collected after the age of 24 hours, although the best “window” for testing is 48 to 72 hours of age. Blood is spotted onto filter paper, allowed to dry, and eluted into a buffer for TSH analysis. This method detects primary CH more effectively than primary T_4_ screening. Primary T_4_ screening with confirmatory TSH testing entails a risk of missing some cases of mild forms of primary CH but can detect some cases of central CH (CCH). Screening strategies for the detection of CCH are based on two approaches: 1) a combination of primary T_4_ and primary TSH screening; and 2) a combination of primary T_4_ screening with secondary TSH testing followed by T_4_ binding protein determination. The inclusion of T_4_ binding protein determinations decreases the number of false positives. The criteria defining a positive result must be adapted to the target disease definition and the resources of the screening program (1|⊕⊕⊕).

#### 1.2.1–1.2.2 Evidence

The most convincing justification for expanding neonatal screening for CH to every country in the world is that this approach is the most effective way of preventing mental retardation and ensuring normal IQ in this patient population (12, 13). Furthermore, because iodine deficiency is the most common preventable cause of mental retardation, developmental disabilities, and CH worldwide (8, 14, 15), neonatal screening for CH can be used as a sensitive indicator of neonatal and maternal iodine nutritional status (16). The strategy for selecting neonatal screening tests focuses on detecting the more severe forms of CH as early as possible because disability due to primary CH is greatest in patients not treated before the age of 3 months (8, 17). TSH screening is the most sensitive test for primary CH detection and should be the single most important test in any screening program (8, 17, 18). Predictably, an increase in the reported incidence of primary CH occurs when cutoff levels for TSH are lowered (3, 12, 17, 19, 20). Studies on long-term outcome are required to determine whether there is a risk of permanent disability in these milder cases with only moderate TSH elevation and normal T_4_ levels and whether these individuals have permanent or transient thyroid dysfunction (6).

There is some published evidence to suggest that neonatal screening for CCH may also fulfil criteria for disease screening (21–24): 1) CCH is a relatively frequent disease with an incidence similar to that of phenylketonuria in some populations; 2) screening tests are available and inexpensive; 3) treatment is available and effective; and 4) the risks of an unfavorable outcome in cases of delayed diagnosis are well known, although outcome studies showing that screening is superior to detection through clinical presentation are lacking (22).

### 1.3 Screening in special categories of neonates at risk of CH

#### Recommendations

1.3.1 Specific biochemical criteria should be used for screening special categories of neonates at risk of transient and permanent CH and in whom initial screening tests may be inappropriate or provide normal results. A strategy of second screening may be required in the following conditions: preterm neonates with a gestational age (GA) of less than 37 weeks; LBW and VLBW neonates; ill and preterm neonates admitted to NICU; specimen collection within the first 24 hours of life; and multiple births, particularly in cases of same-sex twins. The repeat specimen should be collected at about 2 weeks of age, or 2 weeks after the first screening test was carried out. The interpretation of screening results should take into account the results of all specimens analyzed in a multiple sampling strategy. The criteria defining a positive screening test result should be adapted for the analytical parameters measured, the method used, and the age at sampling and maturity (GA/birth weight) of the infant (2|⊕⊕○).

#### 1.3.1 Evidence

Several studies have generated data that argue for multiple sampling in preterm neonates with a GA of less than 37 weeks, LBW and very LBW neonates, ill and preterm neonates admitted to NICU, infants from whom a specimen is collected within the first 24 hours of life, and neonates from multiple births, particularly in case of monozygotic twins (8, 9, 12, 17, 19, 25–29). This approach reflects concern that primary CH may be masked in these situations due to the suppression of TSH caused by drug administration (30, 31), by hypothalamic-pituitary immaturity (32), by fetal blood mixing in multiple births (33), and by other effects of serious neonatal illnesses (34–36). Thus, it is the policy in many centers to remeasure dried blood spot (DBS) TSH in at-risk infants as they approach discharge from hospital. Repeat screening has not been adopted by all screening programs, some centers arguing that the limited data available suggest that, although it has identified neonates with delayed rise in TSH, this is mostly a transient problem (27). Further outcome data from this complex population of newborn infants are needed to better inform clinical practice.

Some neonatal screening programs test for T_4_ alone in the initial screen, whereas TSH may also be assessed on the first specimen. However, most programs initially assessing T_4_ only subsequently evaluate TSH concentration for the neonates with the lowest T_4_ values (usually the lowest 10% of the T_4_ values for the day). If TSH concentration is high, the infant is recalled for evaluation and testing. Repeat DBS specimens are collected if the T_4_ value is below a defined cutoff value for GA (37, 38). If the TSH test result in the initial screening was normal, but repeat testing shows TSH concentration to be high, the evaluation and possible treatment of transient (in most cases) or permanent primary CH should be initiated promptly. Neonates with a persistently low T_4_ concentration in DBS tests should have serum FT4 and TSH determinations to confirm or exclude CCH (8, 9).

## 2.0 Criteria for Diagnosis

### 2.1 Biochemical criteria for use in the decision to initiate treatment in an infant with high TSH and/or low FT4 concentration

#### Recommendations

2.1.1 We recommend starting treatment immediately after baseline serum TSH and FT4 determination if DBS TSH concentration is ≥ 40 mU/L of whole blood. If DBS TSH concentration is < 40 mU/L of whole blood, the clinician may postpone treatment, pending the serum results, for 1–2 days (1|⊕⊕○).

2.1.2 We recommend starting treatment immediately if serum FT4 concentration is below the norm for age, regardless of TSH concentration (1|⊕⊕⊕).

2.1.3 We suggest that treatment should be started if venous TSH concentration is persistently > 20 mU/L, even if serum FT4 concentration is normal (2|⊕○○).

2.1.4 When venous TSH concentration is between 6 and 20 mU/L in a well baby with an FT4 concentration within the normal limits for age, we suggest diagnostic imaging to try to establish a definitive diagnosis (2|⊕○○). If TSH concentration remains high for more than 3 to 4 weeks, we suggest (in discussion with the family) either starting L-T_4_ supplementation immediately and retesting, off treatment, at a later stage; or retesting 2 weeks later without treatment (2|⊕○○).

#### 2.1.1- 2.1.4 Evidence

L-T_4_ treatment must be started immediately if venous FT_4_ or TT4 levels are low, given the known adverse effect of untreated decompensated CH on somatic growth and neurodevelopment (39). Previous work has shown that the likelihood of decompensated hypothyroidism is high if DBS TSH values are above 40 mU/L (40), justifying immediate treatment if DBS TSH concentration is above this value. Given that the period between birth and the age of 3 years is a critical time for neurocognitive development, most clinicians would advocate treatment when TSH concentration is > 20 mU/L, carefully monitoring thyroid function to avoid overtreatment, and retesting after 3 years if the thyroid is normally located (41). Management remains a matter of debate for cases in which TSH concentrations are high, but to a lesser extent (6–20 mU/L), and FT4 levels are normal (42). The family should be informed that this is a “gray area” but that many clinicians would advise “playing safe” and treating during early childhood in this situation (43).

### 2.2 Communication of high TSH concentration results on neonatal screening to the family, the family doctor, and the local pediatrician

#### Recommendations

#### Communicating the initially high capillary TSH concentration result

2.2.1 The detection of a high TSH concentration on screening should be communicated by an experienced person (2|⊕○○), such as a member of the screening laboratory staff or one of the pediatric endocrine team.

2.2.2 The high TSH concentration should be communicated to the family either by telephone or in person as soon as possible (2|⊕○○).

2.2.3 Communication may be directly with the family or via the family doctor, health visitor, or midwife (2|⊕○○).

#### Seeing the baby for clinical examination, investigation, and treatment

2.2.4 If possible, the baby should be seen for clinical assessment and venous thyroid function testing on the day of referral or on the next day at the latest (1|⊕○○).

2.2.5 Centers should have relevant information materials about the diagnosis and management of CH in the appropriate languages for their community (2|⊕○○).

2.2.6 The parents should be shown how to give the first dose of L-T_4_, by either the clinician or the pharmacist (2|⊕○○).

#### Communication with the family doctor, local pediatrician, and educators

2.2.7 The baby's family doctor and local pediatrician should be notified either by telephone or by letter to outline the provisional diagnosis and management (1|⊕○○).

2.2.8 Options regarding shared care should be discussed with the clinician at the local level (1|⊕○○).

2.2.9 When the child reaches nursery or school age, the consensus group advises against informing educators and teachers about the child having CH, to prevent “labeling” and stigmatization (2|⊕⊕○).

#### 2.2.1–2.2.9 Evidence

ESPE guidelines provide little information about contacting and counseling the families of neonates with high TSH concentrations (44). A survey of British pediatricians in 2006/2007 indicated that 54 of the 119 pediatricians questioned (43%) would always see the infant on the day of notification, whereas 65 (55%) would usually see the baby a day or so later (45). Age at treatment initiation during the first month of age was not shown to be a factor in educational attainment in a large French cohort study (46), but there are common sense grounds for starting L-T_4_ treatment as soon as possible after birth to prevent irreversible neurocognitive impairment.

### 2.3 Criteria for assessing CH severity in terms of clinical, biochemical, and radiological features

#### Recommendations

2.3.1 CH severity can be assessed clinically—on the basis of symptomatic hypothyroidism; biologically—as severe, moderate, or mild on the basis of serum FT4 levels of <5, 5 to <10, and 10 to 15 pmol/L, respectively; on the basis of delayed epiphyseal maturation on knee x-ray; and in terms of the etiology of CH (1|⊕⊕⊕).

A serum thyroglobulin concentration below the detection threshold is highly suggestive of athyreosis or a complete thyroglobulin synthesis defect (2|⊕⊕⊕).

#### 2.3.1 Evidence

The clinical symptoms and signs of symptomatic CH include sleepiness, not waking for feeds, poor and slow feeding, cold extremities, prolonged neonatal jaundice, lethargy, hypotonia, macroglossia, umbilical hernia, and dry skin with or without a coarse/puffy face. Persistence of the posterior fontanelle, a large anterior fontanelle, and a wide sagittal suture all reflect delayed bone maturation, which can be further documented by knee x-ray. The absence of one or both knee epiphyses has been shown to be related to: 1) T_4_ concentration at diagnosis; and 2) IQ outcome, and is thus a reliable index of intrauterine hypothyroidism (47, 48). A threshold effect of subnormal TT4 concentration on IQ has also been claimed, with a 10-point IQ difference between children with initial TT4 concentrations below 40 nmol/L (equivalent to 5.5 pmol/L of FT4) and children with normal TT4 concentrations (49). Data for 82 10-day-old neonates yielded 2.5th percentile, median, and 97.5th percentile values of 1.18, 1.75, and 2.49 ng/dL—equivalent to 15.2, 22.5, and 32 pmol/L (50)—making it possible to construct a scale of biochemical severity on the basis of plasma FT4 concentrations of <5, 5 to <10, and 10 to 15 pmol/L. Imaging may reveal severe primary CH in cases of absence/severe hypoplasia of the gland or of complete organification defect with goiter. Alternatively, imaging may show various degrees of severity in cases of ectopic gland or normally shaped and located gland. A pragmatic conclusion as to the severity of CH can therefore be made when the clinical history, physical findings, initial venous blood biochemistry results, and knee x-ray and thyroid imaging results (if available) are considered together.

### 2.4 The place of imaging techniques—scintigraphy with or without perchlorate discharge test and ultrasonography—in the diagnosis of CH

#### Recommendations

2.4.1 We recommend performing imaging studies to determine the specific etiology (1|⊕⊕○).

2.4.2 Both scintigraphy and ultrasound should be considered in neonates with high TSH concentrations (2|⊕⊕○).

2.4.3 Imaging should never be allowed to delay the initiation of treatment. Scintigraphy should be carried out within 7 days of starting L-T_4_ treatment (1|⊕⊕○).

2.4.4 Infants found to have a normal-sized gland in situ in the absence of a clear diagnosis should undergo further reassessment of the thyroid axis at a later age (1|⊕○○).

#### 2.4.1–2.4.4 Evidence

#### Scintigraphy

Scintigraphy may be carried out with either 10–20 MBq of technetium-99m (^99m^Tc) or 1–2 MBq of iodine-123 (^123^I). ^99m^Tc is more widely available, less expensive, and quicker to use than ^123^I. However, ^123^I is specifically taken up by the thyroid gland and gives a clearer scan than ^99m^Tc (51). Scintigraphy can identify athyreosis (absence of uptake), hypoplasia of a gland in situ (with or without hemithyroid), a normal or large gland in situ with or without abnormally high levels of uptake, and an ectopic thyroid at any point along the pathway of the normal embryological descent from the foramen caecum at the base of the tongue to the thyroid cartilage. An enrichment of the tracer within the salivary glands can lead to misinterpretation, especially on lateral views, but this can be avoided by allowing the infant to feed before scintigraphy, thus emptying the salivary glands. When the thyroid is in the normal position, a discharge of >10% of the ^123^I dose when perchlorate is administered at 2 hours (the perchlorate discharge test) indicates an organification defect (51). Scintigraphy may show no uptake despite the presence of a eutopic thyroid gland with excess iodine intake through exposure (eg, from antiseptic preparations), maternal TSH receptor blocking antibodies, TSH suppression from L-T_4_ treatment, and inactivating mutations in the TSH receptor and the sodium/iodide symporter (NIS) (52, 53).

#### Ultrasound

The thyroid gland is a superficial structure that can be imaged by ultrasonography with a high-frequency linear array transducer (10–15 MHz) at a resolution of 0.7 to 1.0 mm. Ultrasound imaging, performed in the longitudinal and axial planes, can be used to investigate the absence or presence, size, echogenic texture, and structure of a thyroid gland in situ. However, it cannot always detect lingual and sublingual thyroid ectopy (54–56), although the use of color Doppler facilitates the identification of thyroid tissue by demonstrating marked increases in blood flow (57). Ultrasonography is highly observer-dependent, and investigators should be particularly wary of misdiagnosing nonthyroidal tissue in the thyroid fossa as a dysplastic thyroid gland in situ (54, 58). Thyroid tissue is more echogenic than muscle but less echogenic than fat. In the absence of thyroid tissue in the normal location, small hyperechoic structures of approximately the same echogenicity as fat are found laterally on both sides of the trachea, mimicking the appearance of a thyroid gland. Cysts have also been described within the empty thyroid area (59).

#### Scintigraphy and ultrasound combined

Combining scintigraphy and thyroid ultrasound in the individual patient helps to: 1) improve diagnostic accuracy (55, 60); 2) identify a eutopic gland, which may be normal, enlarged, or hypoplastic, thus guiding further diagnostic investigations, including molecular genetics studies; 3) prevent the incorrect diagnosis of athyreosis in the context of an absence of uptake on scintigraphy when ultrasound shows a normal gland in situ; and 4) detect thyroid ectopy reliably. Table 1 shows the diagnostic patterns to be found in thyroid dysgenesis, dyshormonogenesis, and some forms of transient CH when ultrasound, scintigraphy, and serum thyroglobulin measurement are combined.

**Table 1.** Thyroid Ultrasound, Scintigraphy, and Serum Thyroglobulin Findings in Thyroid Dysgenesis, Dyshormonogenesis, and Some Forms of Transient CH

Defect	Thyroid Ultrasound	Thyroid Scintigraphy	Serum Thyroglobulin Concentration
Thyroid dysgenesis			
Apparent athyreosis	No thyroid tissue seen	No uptake	Detectable (≥2 μg/L)
True athyreosis	No thyroid tissue seen	No uptake	Undetectable
Ectopy	Either no thyroid tissue seen or ectopic tissue seen (especially if in a sublingual or perihyoid location)	Uptake into ectopic gland	Usually ↑ but may be N or ↓
Hypoplasia in situ	Small eutopic gland	Low level of uptake in a normally sited gland	N or ↓
Hemiagenesis	Hemithyroid	Hemithyroid	N
Dyshormonogenesis			
NIS/SCL5A5	Enlarged gland	Uptake absent or ↓↓	↑
Thyroid peroxidase, TPO	Enlarged gland	High level of uptake; positive perchlorate discharge test	↑↑
Dual oxidase 2, DUOX2/dual oxidase 2 maturation factor, DUOXA2	Enlarged gland	High level of uptake; positive perchlorate discharge test	↑
Thyroglobulin, TG	Enlarged gland	Avid uptake; normal perchlorate discharge test	↓↓ or undetectable
Pendred syndrome, pendrin PDS/SCL26A4	Normal/enlarged gland	High level of uptake; positive perchlorate discharge test	↑
Dehalogenase, IYD/DEHAL1	Enlarged gland	Avid uptake; normal perchlorate discharge test	↑
Transient CH			
Acute iodine excess	Normal gland *in situ*	No uptake	N or ↓
Chronic iodine deficiency	Large gland	Avid uptake	↑
Maternal blocking antibodies	N or small gland	Uptake ↓ or absent	N or ↓
TSH receptor, +/−	N or small gland	Uptake ↓ or absent	N or ↓

Abbreviation: N, normal.

### 2.5. Congenital malformations and syndromes that should be systematically sought for in infants with CH

#### Recommendations

2.5.1 A thorough physical examination should be carried out in all neonates with high TSH concentrations for the detection of congenital malformations, particularly those affecting the heart, and in children for the identification of any underlying dysmorphic syndrome or neurodevelopmental disorders (1|⊕⊕⊕).

#### 2.5.1 Evidence

The prevalence of congenital malformations, particularly cardiac malformations, including septal defects, renal abnormalities, and the risk of neurodevelopmental disorders is higher in subjects with CH than in the general population (61–65). However, care must be taken to distinguish between true CH and transient increases in TSH concentration in sick infants with and without extrathyroidal malformations, including heart and great vessel defects such as patent ductus arteriosus (66). There is no good evidence to suggest that additional screening measures, other than careful clinical examination, are required for detecting extrathyroidal malformations or comorbidities.

Down syndrome is associated with a mild increase in TSH concentration from the neonatal period onward, although it is usually too small for detection by neonatal screening, as well as a shift to the left of the FT4 distribution so that mean values are lower when compared with the general population (67). Pendred syndrome, with or without goiter, and pseudohypoparathyroidism may both present with mild or moderate increases in TSH concentration during the neonatal period and should be included in the differential diagnosis of CH with gland in situ (60).

## 3.0 Treatment and Monitoring of CH

### 3.1 Initial treatment and therapeutic regimens

#### Recommendations

3.1.1 L-T_4_ alone is recommended as the treatment of choice for CH (1|⊕⊕○).

3.1.2 Treatment with L-T_4_ should be started as soon as possible, and no later than the first 2 weeks of life or immediately after confirmatory serum test results in infants identified in a second routine screening test. We recommend an initial L-T_4_ dose of 10 to 15 μg/kg per day. Infants with severe disease, as defined by a very low pretreatment TT_4_ or FT4 concentration, should be treated with the highest initial dose, and those with a mild to moderate hypothyroidism with a lower dose (1|⊕⊕○).

3.1.3 L-T_4_ should be administered orally. If oral administration is not possible, it can also be administered iv, in which case the iv dose should be no more than 80% the oral dose. The dose should then be adjusted according to TSH and FT4 determinations (1|⊕⊕○).

3.1.4 We recommend the administration of L-T_4_ in tablet form. In neonates and infants, the tablets can be crushed and administered via a small spoon, with suspension, if necessary, in a few milliliters of water or breast milk. L-T_4_ can also be administered in liquid form, but only if pharmaceutically produced and licensed L-T_4_ solutions are available (1|⊕⊕○). A brand rather than a generic formulation is recommended in CH, at least in infancy (2|⊕⊕○).

3.1.5 L-T_4_ can be administered or taken in the morning or evening, either before feeding or with food, but it should be administered in the same way every day. The dose should then be adjusted according to TSH and FT4 determinations to establish the appropriate dose in each setting. Caution should be taken with the administration of vitamin D during the first weeks of life, and the intake of soy, iron, and calcium at the time of L-T_4_ administration should be avoided (1|⊕⊕⊕).

3.1.6 Parents should be provided with written instructions on L-T_4_ treatment to avoid uncertainties that might hinder compliance (1|⊕○○).

#### 3.1.1–3.1.6 Evidence

T_3_ is the biologically active hormone, but there is no evidence that combined therapy with L-T_4_ and L-T_3_ is more beneficial than treatment with L-T_4_ alone, probably due to the high degree of efficiency of endogenous deiodinases, which break T_4_ down into T_3_ (68, 69). L-T_4_ is available in tablet form (the most widely used form) or as a pharmaceutically produced and licensed liquid form. Unlike suspensions prepared by pharmacists, this licensed L-T_4_ solution allows reliable dosing and is a convenient way of administering L-T_4_, particularly to infants and young children (70–73). Recent evidence suggests that brand and generic L-T_4_ are not bioequivalent and that for CH, particularly in severe cases, it is prudent to use a brand preparation (74).

The orally administered hormone has a mean bioavailability of 50 to 80%, which may be influenced by the presence of food (soy) or minerals (calcium, iron). In infants with CH, hypersensitivity to vitamin D administration with hypercalcemia has been described during the first few weeks of L-T_4_ treatment, possibly due to the administration of prophylactic doses of vitamin D (75, 76). In adults, L-T_4_ administration at bedtime seems to be even more effective in terms of thyroid hormone levels than administration in the morning and is now considered to be as effective as morning administration in the fasting state (77).

The early initiation of L-T_4_ treatment, within the first 2 weeks of life, has been shown to be crucial for neurodevelopment and for the achievement of a normal intellectual outcome in affected children (78–81). Disease severity, as judged by very low initial levels of thyroid hormones due to the absence or loss of function of the thyroid gland and by severely delayed bone age, has also been shown to be an important predictive factor for neurodevelopment (49, 82–87). Severely affected children may benefit from a higher initial dose of L-T_4_, leading to the more rapid normalization of thyroid hormone levels and potentially resulting in a better intellectual outcome (88–91).

### 3.2 Monitoring of treatment and adverse events

#### Recommendations

3.2.1 The monitoring of L-T_4_ treatment should be based on periodic measurements of serum or plasma FT4 (or TT_4_) and TSH concentrations. The blood samples for laboratory evaluation should be collected at least 4 hours after the last L-T_4_ administration (1|⊕⊕○). We recommend maintaining TSH in the age-specific reference range (but to avoid undetectable TSH < 0.05 mU/L) and serum concentrations of TT_4_ or FT4 in the upper half of the age-specific reference range (1|⊕⊕○). If necessary, the treatment should be adjusted according to the hormone concentrations measured, but decreases in LT4 dose should not be based on a single high FT4 concentration during treatment (1|⊕⊕○). Clinicians should be familiar with the reference ranges for the methods used by the laboratory carrying out the tests (1|⊕○○).

3.2.2 We recommend performing the first follow-up examination 1 to 2 weeks after the start of L-T_4_ treatment, with intense follow-up over the first year of life (every 2 weeks until TSH levels are completely normalized and every 1 to 3 months thereafter, until the age of 12 months). Between the ages of 1 and 3 years, children should undergo frequent clinical and laboratory evaluations (every 2 to 4 months), with regular evaluations every 3 to 12 months thereafter until growth is completed. Measurements should be performed at more frequent intervals if compliance is questioned or abnormal values are obtained, and 4 to 6 weeks after any change in L-T_4_ dose or L-T_4_ formulation (eg, switch from brand to generic L-T_4_) (1|⊕○○).

3.2.3 The incidence of adverse events during L-T_4_ treatment is very low. The careful monitoring of thyroid hormone parameters, during both initial and maintenance treatment, is recommended to minimize the risk (1|⊕○○).

3.2.4 In children with pre-existing cardiac insufficiency, we suggest introducing L-T_4_ at 50% of the target replacement dose and increasing this in accordance with FT4 levels after 2 weeks (2|⊕○○).

#### 3.2.1–3.2.4 Evidence

The rapid normalization of thyroid hormone levels (within the first 2 weeks after treatment initiation) and the maintenance of relatively higher FT4 concentrations during the first year of life lead to a better intellectual outcome (90, 92, 93). The frequent monitoring of TSH and FT4 levels is required for this and also for preventing the occurrence of prolonged periods of supraphysiological thyroid hormone levels (94–96). After adjustment to L-T_4_ dosage, it is appropriate to recheck thyroid function, and the recommended interval of 4–6 weeks is in keeping with the American Academy of Pediatrics guidelines (8). The adequate treatment of CH minimizes the risk of treatment-related adverse effects (97–99). Based on neurological and cardiac complications that have occasionally been described (albeit primarily in patients with inadequate T_4_ treatment), patients with pre-existing health conditions or with a very late diagnosis may require special attention during treatment (100, 101).

### 3.3. Criteria for re-evaluating the thyroid axis, to distinguish between permanent CH and transient increases in TSH concentration, and for treatment withdrawal in children with normally sited gland

#### Criteria for re-evaluation of the thyroid axis

#### Recommendations

3.3.1 We recommend re-evaluation of the thyroid axis in cases in which no etiological diagnostic assessment was carried out during early infancy and/or when treatment was started in the context of the infant being ill (eg, preterm). Re-evaluation is also mandatory when initial evaluation has shown a normally located gland, with or without goiter, in neonates with positive thyroid antibodies, in children who have required no increase in L-T_4_ dose since infancy, and in children in whom no enzyme defect has been identified, either because no molecular genetic investigations have been carried out or because investigations have proved negative for all mutations tested (1|⊕⊕○).

3.3.2 Retesting off treatment may be waived if venous TSH concentration has risen after the first year of life, due to either L-T_4_ underdosage or poor compliance with treatment (1|⊕⊕○).

3.3.3 Re-evaluation of the thyroid axis is not indicated when thyroid dysgenesis has been conclusively shown on imaging or (with the exception of DUOX.2 mutations or Pendred syndrome) when dyshormonogenesis has been confirmed by molecular genetic testing (1|⊕⊕⊕).

#### 3.3.1–3.3.3 Evidence

Thyroid re-evaluation is important if no definitive diagnosis was made during the neonatal period; it has been shown that one-third of patients with CH and normally located glands may have transient thyroid dysfunction (41, 60). Re-evaluation is unnecessary if thyroid imaging at the time of neonatal screening showed thyroid ectopy, apparent athyreosis, or true athyreosis. However, caution is required when diagnosing athyreosis on the basis of isotope scanning alone because an absence of uptake in the context of a gland normally located on ultrasound scan may occur with excess iodine exposure, maternal antibodies blocking the TSH receptor, or NIS gene defects (52, 53).

Re-evaluation is essential in subjects who were preterm or sick during the neonatal period (27). Other infants in whom initial evaluation showed a normal/slightly small gland on ultrasound with little or no uptake on scintigraphy should also be re-evaluated because this pattern is suggestive of either maternal antibodies blocking the TSH receptor or biallelic-TSH receptor mutations (102). Because iodine deficiency may mimic dyshormonogenesis (both conditions display thyroid enlargement and avid tracer uptake), retesting is indicated in children who appear to have mild dyshormonogenesis (103). Transient CH has also been linked to genetic defects such as heterozygous DUOX2 mutation (104, 105).

#### Timing of thyroid re-evaluation

#### Recommendations

3.3.4 Re-evaluation of the thyroid axis, off treatment, should normally take place after the age of 3 years (1|⊕⊕○).

3.3.5 Earlier re-evaluation may be indicated if the clinical context renders transient increases in TSH concentration highly probable; for example: 1) in the case of newborns in whom thyroid peroxidase or TSH receptor antibodies are detectable in the blood; and 2) when a eutopic, normally sized gland is found on ultrasound scans (2|⊕⊕○).

#### 3.3.4–3.3.5 Evidence

Magnetic resonance imaging studies have shown that the myelination of the central nervous system is completed by 36 to 40 months of age (106), at which point the child is more likely to be co-operative for thyroid imaging than at the age of 1 or 2 years. However, if transient increases in TSH concentration are likely, the clinician may consider the earlier withdrawal of treatment from 1 year of age.

#### Method of thyroid re-evaluation

#### Recommendations

3.3.6 If a precise diagnosis is sought, L-T_4_ should be phased out over a 4 to 6-week period (depending on the size of the maintenance dose) and a full re-evaluation carried out at the end of this period, with biochemical testing and thyroid imaging if hypothyroidism is confirmed (2|⊕○○).

3.3.7 If the clinician wishes to establish the presence or absence of primary hypothyroidism rather than to obtain an exact diagnosis, L-T_4_ dose may be decreased by 30% for 2 to 3 weeks. If an increase in TSH concentration to ≥ 10 mU/L is observed during this period, then continuing hypothyroidism can be assumed. By contrast, if thyroid function remains normal, the dose may be reduced further, followed by retesting (2|⊕⊕○).

#### 3.3.6–3.3.7 Evidence

By the age of 2 to 3 years, the severity of thyroid impairment may be evident from a lower dose requirement (41, 103) or, if compliance has been imperfect, high TSH levels despite treatment.

### 3.4 Treatment and monitoring in pregnant women with CH

#### Recommendations

3.4.1 For women with CH who are planning a pregnancy, even greater efforts should be made to ensure that maternal thyroid hormones are optimal. In newly pregnant patients, we recommend an immediate increase in L-T_4_ dose by 25 to 30% after a missed menstrual cycle or positive home pregnancy test (1|⊕○○).

3.4.2 TSH and FT4 (or TT_4_) levels should be evaluated as soon as pregnancy is confirmed, every 4 to 6 weeks during the pregnancy, and 4 weeks after every change in dose (1|⊕⊕⊕).

3.4.3 The goal of the treatment is to maintain TSH concentration below 2.5 mU/L in the first trimester and below 3 mU/L later in pregnancy (1|⊕○○).

#### 3.4.1–3.4.3 Evidence

There is currently no specific evidence for treatment schedules in pregnant women with CH. We therefore refer to the guidelines of the American Thyroid Association and The Endocrine Society regarding the management of thyroid disease during pregnancy (107–109).

## 4.0 Outcomes of Treated Patients

### 4.1 Neurodevelopmental outcome

#### Neurocognition, behavior, memory, psychomotor, school progression, language, hearing, and visuospatial skills

#### Recommendations

4.1.1 Psychomotor and language development as well as school progression should be monitored and recorded in all children with CH (1|⊕⊕○). Clinicians should pay particular attention to developmental delays or learning difficulties and to attention problems in children with severe CH (athyreosis, absent knee epiphyses at term, very low T_4_ and very high TSH concentrations at diagnosis) or poor endocrine control, particularly during the first year, and in those from economically disadvantaged families (1|⊕⊕⊕).

4.1.2 We suggest specialized stimulation of motor development, if required, and a personalized educational plan if school progression is affected (2|⊕⊕○).

4.1.3 Memory deficits may be corrected by targeted training (2|⊕⊕○).

4.1.4 Concerns about behavior should be addressed from the time of diagnosis until school age (2|⊕⊕○).

4.1.5 Adequate treatment throughout childhood is essential, and overtreatment should be avoided (1|⊕⊕⊕).

4.1.6 The determinants of remaining deficits require further study.

#### 4.1.1–4.1.6 Evidence

With early and adequate treatment, intellectual disability (defined as an IQ < 70) has disappeared from cohorts screened for CH, and the mean global IQ is now 10 to 30 points higher in these patients than in the prescreening era (1). Some affected patients still have neurocognitive and behavioral sequelae of CH that persist into adolescence and adulthood and that are related to disease severity (78, 82, 110, 111). Cognitive outcome is related to age at treatment and L-T_4_ dose (90); school progression may be affected (112). Cognitive outcome is related to the parents' socioeducational status (49).

Behavior scores on initial admission to school are within the normal range, (89) and the perception of the impact of CH on behavior varies with age and differs between children and their parents (113). The impact of informing teachers of the diagnosis of CH has not been investigated. Patients with CH have no increase in the risk of attention deficit-hyperactivity disorder but may have more sustained attention problems related to episodes of overtreatment (114, 115) and, in severe cases, slower information processing (116).

Subtle and specific memory deficits and reduced hippocampal volumes may be observed (117). There is also a risk of fine motor impairment (118). However, most early-treated patients are well integrated into society with no impairment in educational level (46).

#### Hearing, visual, verbal development

#### Recommendations

4.1.7 Repeated (not just neonatal) hearing tests should be carried out before school age and as required (2|⊕⊕○).

4.1.8 Assessing patients for evidence of visual processing problems (not just visual acuity) is suggested (2|⊕○○).

4.1.9 We recommend screening for delays in speech acquisition by the age of 3 years and propose speech therapy as required (2|⊕⊕○).

#### 4.1.7–4.1.9 Evidence

Even after excluding patients with Pendred syndrome, a higher prevalence of hearing impairment has been observed in patients with CH than in the reference population, possibly necessitating the use of hearing aids in childhood. Substantial adverse effects on speech development, school performance, and social interactions may occur if hearing impairment is undiagnosed (119). This impairment may result from the role of thyroid hormone in cochlear development and auditory function (46, 120, 121). There is also a risk of visual processing problems (10, 46).

### 4.2 Health-related quality of life

#### Recommendations

4.2.1 Compliance with treatment should be promoted throughout life (1|⊕⊕⊕).

4.2.2 In future studies of HrQOL, “focusing illusion” should be considered (a form of stigmatization similar to “labeling” at school) (2|⊕○○).

#### 4.2.1–4.2.2 Evidence

There is a risk of a subtle decrease in HrQOL, particularly in mental dimensions and if treatment is suboptimal (46, 122, 123).

### 4.3 Patient and professional education; compliance/adherence

#### Recommendations

4.3.1 Medical education about CH should be improved at all levels, with regular updates (1|⊕⊕⊕).

4.3.2 We suggest identifying resources in the community to which patients can be referred for continuing education about self-management of their condition and to update their knowledge of CH. The education of both parents and patients is essential, with particular attention paid to the transition to adult care and management during pregnancy (1|⊕⊕⊕).

#### 4.3.1–4.3.2 Evidence

The adherence of physicians to guidelines is low (45). Poor adherence with treatment and low treatment adequacy are prevalent at all ages (46, 124).

### 4.4 Growth, puberty, and fertility

#### Recommendations

4.4.1 Adherence to treatment influences growth and should be promoted (1|⊕⊕⊕).

4.4.2 Provided treatment is adequate, it is appropriate to provide patients and their parents with reassurance about growth, puberty, and fertility (1|⊕⊕⊕).

#### 4.4.1–4.4.2 Evidence

Length and height increase within normal limits in patients with adequately treated CH (125). Patients may be overweight in early childhood and adulthood (46, 126). Head circumference may be greater than normal, but this reflects bone rather than brain development (127). The onset of puberty, age at menarche, and menstrual cycles are normal. Fecundity is generally normal, except in the most severely affected female patients (128, 129).

### 4.5 Bone health

#### 4.5.1 Recommendations

We recommend that patients with CH should be adequately treated with L-T_4_ and consume 800–1200 mg of calcium daily, ideally from dietary sources, with calcium supplements added if dietary calcium intake is insufficient (2|⊕○○).

#### 4.5.1 Evidence

Thyroid hormones exert major effects on bone remodeling. Patients overtreated with L-T_4_ display higher levels of bone resorption than of bone formation, leading to progressive bone loss. The goal of therapy is to render the patient euthyroid, with a normal TSH concentration. Only a few studies have evaluated the impact of long-term L-T_4_ treatment on bone mineral density. Two studies reported that bone mineral density in children and young adults with CH is within the normal range (130, 131). More data for patients treated with the currently used doses are required.

### 4.6 Metabolic and cardiovascular health

#### Recommendations

4.6.1 Weight should be closely monitored. Lifestyle interventions, including diet and exercise, should be encouraged in individuals with CH (2|⊕○○).

4.6.2 Optimal treatment of CH is essential for cardiovascular health (1|⊕○○).

#### 4.6.1–4.6.2 Evidence

Patients with CH have a higher risk of being overweight and, thus, of metabolic complications (46). In addition to the higher risk of congenital heart malformations (61, 62), there is a subtle increase in cardiovascular risk factors in young adults with CH, related to treatment adequacy (132).

## 5.0 Genetic Counseling and Antenatal Management

### 5.1 Criteria for genetic counseling

#### Recommendations

5.1.1 Genetic counseling should provide information about the risk of recurrence of CH in a family with CH, based on family history and thyroid morphology. Each family with an affected child should have access to information on the two major forms of CH (dysgenesis and dyshormonogenesis) and should receive an explanation of inheritance and recurrence rate. A certified geneticist or a genetic counselor (depending upon the organization of healthcare in the country concerned) should be made available in some cases. In such cases, given the current state of knowledge, we propose targeted rather than general genetic counseling in the context of certain clinical situations, described in Table 2 (2|⊕⊕○).

**Table 2.** Situations in Which Genetic Counseling Should Be Offered

I. Pregnant women
Positive family history for nonsyndromic CH
Dyshormonogenesis (previously affected child) (1|⊕⊕⊕)
Dysgenesis (at least 1 member of the family) (2|⊕⊕○)
Positive family history of syndromic CH with
Neurological disorders, including unexplained mental retardation
Deafness
Congenital heart disease, surfactant deficiency syndrome
Cleft palate
Kidney malformations
Any sign of Albright hereditary osteodystrophy (GNAS mutation) (1|⊕⊕○)
Unexplained abnormality of T_4_, T_3_, or TSH levels in family members (mild forms of CH) (2|⊕⊕○)
II. Infant or child with CH (2|⊕⊕○)
Subject with
Deafness
Neurological signs (hypotonia, choreoathetosis, intellectual disability)
Lung disorders (surfactant deficiency syndrome, interstitial lung disease)
Congenital heart disease
Cleft palate
Kidney malformations
Any sign of Albright hereditary osteodystrophy (GNAS mutation)
Family history
Consanguinity
Kidney malformations
Deafness
Specific malformations (as listed above)
Unexplained mental retardation despite adequate treatment of CH in family members
Any sign of Albright hereditary osteodystrophy (GNAS mutation)

#### 5.1.1 Evidence

In primary CH, about 80% of cases are due to thyroid dysgenesis and 20% are due to thyroid dyshormonogenesis. Thyroid dyshormonogenesis is caused by mutations in genes encoding proteins involved in thyroid hormone synthesis: the *SCL5A5/NIS* (iodide transport defect; OMIM No. 274400); pendrin, *SCL26A4/PDS* (Pendred syndrome; OMIM No. 274600); thyroglobulin, *TG* (OMIM No. 274700); thyroid peroxidase, *TPO* (OMIM No. 274500); dual oxidase 2, *DUOX2* (OMIM No. 607200); dual oxidase maturation factor 2, *DUOX2A* (OMIM No. 274900); or iodotyrosine deiodinase, *IYD*/*DEHAL1* (OMIM No. 274800). These mutations are inherited in an autosomal recessive manner and are associated with no other malformations, other than deafness in Pendred syndrome (133). Isolated thyroid dysgenesis (OMIM No. 218700) is generally a sporadic disease. However, three observations suggest a possible, as yet unknown genetic basis: 1) a higher rate of familial cases (>15 times higher) than would be expected by chance alone; 2) minor morphological thyroid abnormalities in euthyroid first-degree relatives of patients with thyroid dysgenesis; and 3) a high incidence of associated extrathyroidal malformations (61–63, 134–137). Specific genetic forms of syndromic and nonsyndromic thyroid dysgenesis and TSH resistance may be associated with mutations in the NK2 homeobox 1 (*NKX2–1*, brain-lung-thyroid syndrome; OMIM No. 610978); Forkhead box E1 (*FOXE-1*, Bamforth-Lazarus syndrome; OMIM No. 241850), Paired box gene 8 (*PAX8*; OMIM No. 218700), NK2 homeobox 5 (*NKX2–5*; OMIM No. 225250), TSH receptor (*TSHR*; OMIM No. 275200); and Gs α (*GNAS*, pseudohypoparathyroidism type 1A; OMIM No. 103580) genes (133, 138) (Table 3).

**Table 3.** Genetic Diagnosis to Detect the Individual Molecular Basis of CH

	Thyroid Morphology, as Assessed by Ultrasonography and/or Scintigraphy	Family History	
		Consanguinity or Siblings/Cousins With CH	Parents With CH
Isolated CH	Normally located thyroid with normal perchlorate discharge test	*TSH-R* (if hypoplasia), *TG* (if goiter, low TG level)	*PAX8*
	Normally located thyroid with abnormal perchlorate discharge test (ie, iodide organification defects)	*TPO, DUOX2/DUOXA2* +/− *TG*	
	Normally located thyroid on ultrasonography, with no iodide uptake on scintigraphy	*SCL5A5/NIS*, *TSH-R* (if hypoplasia)	
Syndromic CH			
Deafness	Normally located thyroid	*SCL26A4/PDS*	
Short stature, obesity, hypocalcemia	Normally located thyroid		*GNAS*
Cleft palate, “spiky” hair	Athyreosis (hypoplasia)	*FOXE1* (no mutations described in patients with ectopic or normally sized and sited thyroid gland to date)	
Kidney agenesis or any malformation of the genitourinary tract	Athyreosis, ectopic thyroid gland, normally located thyroid +/− hypoplasia	*PAX8*	*PAX8*
Choreoathetosis or neurological disease	Normally located thyroid, hypoplasia (athyreosis)	*NKX2–1* (no mutations described in ectopic cases so far)	*NKX2–1*
Lung disorders (surfactant deficiency syndrome at term, interstitial lung disease)	Normally located thyroid, hypoplasia (athyreosis)	*NKX2–1* (no mutations described in ectopic cases so far)	*NKX2–1*
Cardiac defects	Ectopy (athyreosis)	*NKX2–5*	*NKX2–5*

### 5.2 Molecular biology in the diagnosis and management of CH

#### Recommendations

5.2.1 Careful phenotypic description of CH patients (including morphological analysis of the thyroid gland) is required, and we suggest that any syndromic association should be studied genetically to identify new genes involved in CH and to ensure that healthcare staff are in a position to offer appropriate genetic counseling. The presence of familial cases of dysgenesis should lead to a search for *TSHR* and *PAX8* gene mutations (2|⊕⊕○).

The genetic diagnosis of CH may facilitate the targeting of specific interdisciplinary follow-up and supportive care for patients (1|⊕⊕○). The relevant features to consider are listed in Table 3.

#### 5.2.1 Evidence

Molecular biology techniques can identify the cause of CH on the basis of family history and thyroid morphology. The identification of a *NKX2–1* mutation implies that special attention should be paid to neurological development and to lung disease in the follow-up of affected children (139, 140). The identification of a *FOXE1* mutation implies that special attention should be paid to neurological development (141). The identification of a *PAX8* mutation should lead to kidney and urinary tract ultrasound and, probably, to the monitoring of renal function if malformations are found (142). The identification of a *SLC26A4/PDS* mutation implies that special attention should be paid to the hearing of the child (143). The identification of a *TG* or *TPO* mutation implies a risk of thyroid cancer within the goiter in adulthood, as demonstrated by long-term follow-up studies in extremely rare published cases (133, 144). It is unclear whether the thyroid cancer is gene-specific or related to goiter development. The identification of a *GNAS* mutation should lead the clinician to focus on other potentially associated endocrine and nonendocrine disorders (145). Despite intensive and focused research, mutations in these genes have been found in fewer than 10% of CH patients to date, and the usual discordance between monozygotic twins (136) remains unexplained.

### 5.3 Potential indications for antenatal diagnosis, screening methods for fetal hypothyroidism, management and criteria for fetal treatment in utero

#### Recommendations

5.3.1 We recommend antenatal diagnosis in cases of goiter fortuitously discovered during systematic ultrasound examination of the fetus, in relation to thyroid dyshormonogenesis (1|⊕⊕⊕); a familial recurrence of CH due to dyshormonogenesis (25% recurrence rate) (1|⊕⊕⊕); and known defects of genes involved in thyroid function or development with potential germline transmission (1|⊕⊕○). Special issues should be considered for syndromic cases with potential mortality and possible germline mosaicism (as for *NKX2–1* gene mutation/deletion and severe pulmonary dysfunction with possible transmission via germline mosaicism). In such circumstances, the discussion of the prenatal diagnosis should be open. The therapeutic management of affected fetuses should comply with the laws in force in the country concerned (1|⊕⊕○). The familial recurrence of CH due to dysgenesis (2% of familial occurrences) requires further study to determine the feasibility and clinical relevance for antenatal detection.

5.3.2 For the evaluation of fetal thyroid volume, we recommend ultrasound scans at 20 to 22 weeks gestation to detect fetal thyroid hypertrophy and potential thyroid dysfunction in the fetus. Goiter or an absence of thyroid tissue can also be documented by this technique. Measurements should be made as a function of GA, and thyroid perimeter and diameter should be measured to document goiter (1|⊕⊕⊕).

5.3.3 We recommend cordocentesis, rather than amniocentesis, as the reference method for assessing fetal thyroid function. Norms have been established as a function of GA. This examination should be carried out only if prenatal intervention is considered (see below) (1|⊕⊕⊕).

5.3.4 In most cases, fetal thyroid function can be inferred from context and ultrasound criteria, and fetal blood sampling is, therefore, only exceptionally required (2|⊕⊕○).

5.3.5 In a euthyroid pregnant woman, a large goiter in the fetus with progressive hydramnios and a risk of premature labor and delivery and/or concerns about tracheal occlusion are criteria in favor of fetal treatment in utero (1|⊕⊕○).

5.3.6 In a hypothyroid pregnant woman, the initial approach should be to treat the pregnant woman, rather than the fetus, with L-T_4_ (1|⊕⊕○).

5.3.7 For goitrous nonimmune fetal hypothyroidism leading to hydramnios, intra-amniotic injections of L-T_4_ have been reported to decrease the size of the fetal thyroid gland. However, experience with this procedure is limited, and the risk of provoking premature labor or infections should be evaluated with care. Thus, follow-up studies are very important to determine the efficacy and possible adverse long-term consequences of medical interventions on the fetus. Such interventions should be performed only by multidisciplinary specialist teams (pediatric endocrinologists, adult endocrinologists for the pregnant mother, neonatologists, and obstetricians with experience in antenatal care and procedures) (1|⊕⊕⊕).

5.3.8 Studies have confirmed the feasibility and safety of intra-amniotic L-T_4_ injection and strongly suggest that this treatment is effective for decreasing goiter size. However, none of the many L-T_4_ regimens used ensures euthyroidism at birth. It is therefore not possible to formulate guidelines from current data. The expert panel proposes the use of 10 μg/kg estimated fetal weight/15 days in the form of intra-amniotic injections. The risks to the fetus and the psychological burden on the parents should be factored into the risk/benefit evaluation (2|⊕○○).

5.3.9 Determination of the indications and optimal modes of prenatal treatment for nonimmune fetal goitrous hypothyroidism will require larger, well-designed studies that would be best conducted via international co-operation between multidisciplinary medical teams. Alternative ways of treating the fetus by administering drugs to the mother should also be investigated (2|⊕○○).

#### 5.3.1–5.3.9 Evidence

Recent advances in fetal imaging techniques (ultrasonography) and fetal hormonology have made it possible to identify thyroid function disorders in the fetus that could potentially be treated in utero by administering drugs to the mother. Several interventions have also been proposed for improving the fetal outcomes of fetal hypothyroid disorders by considering the fetus as the patient to be treated and gaining direct access to the amniotic cavity. These approaches range from public health interventions with clear benefits and negligible risks, such as increasing the iodine intake of all pregnant women, to procedures with a much less clear benefit-to-risk ratio, such as cordocentesis for determining thyroid function in a fetus with goiter and repeated intra-amniotic injections of L-T_4_ (146–152) (Table 4).

**Table 4.** Screening, Prevention, and Management of Fetal Hypothyroidism

Adequate iodine intake should be ensured for all pregnant women (250 μg/d).
For women with a personal or family history of thyroid disease, serum TSH and FT_4_ concentrations should be determined before pregnancy, at the start of pregnancy, and during pregnancy.
On ultrasonography at about 22 and 32 wk gestation, fetal thyroid diameter and circumference should be measured; if above the 95th percentile for GA, a fetal thyroid disorder should be considered.
If a pregnant woman is treated with L-T_4_, care should be taken to ensure an appropriate increase in dose during the course of the pregnancy.
If fetal goiter is documented, cordocentesis and fetal serum FT4 and TSH determinations should be considered, and intra-amniotic L-T_4_ injections should be administered if severe hypothyroidism is diagnosed and progressive hydramnios develops.

## Conclusions

Patients with CH benefit from neonatal screening, which makes it possible to initiate essential replacement therapy immediately. This consensus highlights the need to identify clear cutoff points for CH screening, without increasing the number of false-positive results. However, the results of long-term prospective studies in subjects with false-positive results are not yet available for the formulation of evidence-based recommendations for diagnosis and management. Based on current outcome data, an immediate treatment should be initiated, and it appears necessary to maintain adequate L-T_4_ treatment throughout the lifespan for most patients, with a particular emphasis on treatment in the first years of life and of treatment optimization in pregnant women with CH. Careful neurodevelopmental and neurosensory evaluations should be started early in life and repeated at important critical developmental phases, taking into account disease severity at diagnosis and providing appropriate interventions as required. Subsequent efforts should focus on educating both patients and caregivers to ensure that adequate treatment is continued into adulthood. Future research should aim to improve our understanding of the pathophysiology of this heterogeneous disorder and to determine whether knowledge of the specific defect in thyroid development or function is likely to improve patient care and outcomes.

## Participants of the Congenital Hypothyroidism Consensus Conference Group

Paulina Alexander, Department of Pediatric Endocrinology, and Diabetes, Charite Children's Hospital, Berlin, Germany; Miguel Alvarez, Institute of Neurology and Neurosurgery, Havana, Cuba; Oliver Blankenstein, Department of Pediatric Endocrinology, and Diabetes, Charite Children's Hospital, Berlin, Germany; Alessandra Cassio, Department of Pediatrics, University of Bologna, Bologna, Italy; Mireille Castanet, Department of Pediatrics, University of Rouen, Rouen, France; Graziano Cesaretti, Department of Pediatric Endocrinology, University of Pisa, Pisa, Italy; Tim Cheetham, Department of Paediatric Endocrinology, Newcastle University, Newcastle-upon-Tyne, United Kingdom; Anna Chiesa, Endocrinology Division, Ricardo Gutierrez Children's Hospital, Buenos Aires, Argentina; Carlo Corbetta, Neonatal Screening Laboratory, Ospedale dei Bambini “Vittore Buzzi,” Milan, Italy; Maria E. Craig, Institute of Endocrinology and Diabetes, Sydney Children's Hospital Network and University of Sydney, Sydney, Australia; Thomas P. Foley, Department of Pediatrics, University of Pittsburgh, Pittsburgh, Pennsylvania; Scott Grosse, Atlanta, Georgia; Marlies Kempers, Radboud University Nijmegen Medical Centre, Department of Genetics, Nijmegen, The Netherlands; Claire de Labriolle-Vaylet, UPMC Univ Paris IV and Nuclear Medicine, Hopital Trousseau, Paris, France; Stephen LaFranchi, Department of Pediatrics, Oregon Health and Sciences University, Portland, Oregon; Dominique Luton, Department of Obstetrics and Gynecology, University Diderot Paris 7, Clichy, France; Masanori Minagawa, Division of Endocrinology, Department of Pediatrics, Chiba Children's Hospital, Chiba, Japan; Palany Raghupathy, Department of Child Health, Christian Medical College, Vellore, India; Robert Rapaport, Department of Pediatric Endocrinology, Mount Sinai School of Medicine, New York, New York; Joanne Rovet, Departments of Psychology and Paediatrics, University of Toronto, Toronto, Canada; Diet S. Rustama, Department of Child Health, Padjadjaran University School of Medicine, Bandung, Indonesia; Mariacarolina Salerno, Department of Pediatrics, University “Federico II,” Naples, Italy; Anna Simon, Department of Pediatrics, Christian Medical College, Vellore, India; Gabor Szinnai, Pediatric Endocrinology and Diabetology, University Children's Hospital Basel, University of Basel, Basel, Switzerland; Toshihiro Tajima, Department of Pediatrics, Hokkadio University School of Medecine, Sapporo, Japan; and Paul van Trotsenburg, Department of Paediatric Endocrinology, Academic Medical Center, University of Amsterdam, Amsterdam, The Netherlands.
